# Mass Lead Intoxication from Informal Used Lead-Acid Battery Recycling in Dakar, Senegal

**DOI:** 10.1289/ehp.0900696

**Published:** 2009-05-14

**Authors:** Pascal Haefliger, Monique Mathieu-Nolf, Stephanie Lociciro, Cheikh Ndiaye, Malang Coly, Amadou Diouf, Absa Lam Faye, Aminata Sow, Joanna Tempowski, Jenny Pronczuk, Antonio Pedro Filipe Junior, Roberto Bertollini, Maria Neira

**Affiliations:** 1 Department of Public Health and Environment, World Health Organization, Geneva, Switzerland; 2 Centre Anti-Poison, CHRU Lille, Lille, France; 3 Senegal Country Office, World Health Organization, Dakar, Senegal; 4 Centre Anti-Poison, Dakar, Senegal; 5 Hôpital de Pikine, Pikine, Dakar, Senegal

**Keywords:** battery, children, intoxication, lead, poisoning, recycling, ULAB

## Abstract

**Background and objectives:**

Between November 2007 and March 2008, 18 children died from a rapidly progressive central nervous system disease of unexplained origin in a community involved in the recycling of used lead-acid batteries (ULAB) in the suburbs of Dakar, Senegal. We investigated the cause of these deaths.

**Methods:**

Because autopsies were not possible, the investigation centered on clinical and laboratory assessments performed on 32 siblings of deceased children and 23 mothers and on 18 children and 8 adults living in the same area, complemented by environmental health investigations.

**Results:**

All 81 individuals investigated were poisoned with lead, some of them severely. The blood lead level of the 50 children tested ranged from 39.8 to 613.9 μg/dL with a mean of 129.5 μg/dL. Seventeen children showed severe neurologic features of toxicity. Homes and soil in surrounding areas were heavily contaminated with lead (indoors, up to 14,000 mg/kg; outdoors, up to 302,000 mg/kg) as a result of informal ULAB recycling.

**Conclusions:**

Our investigations revealed a mass lead intoxication that occurred through inhalation and ingestion of soil and dust heavily contaminated with lead as a result of informal and unsafe ULAB recycling. Circumstantial evidence suggested that most or all of the 18 deaths were due to encephalopathy resulting from severe lead intoxication. Findings also suggest that most habitants of the contaminated area, estimated at 950, are also likely to be poisoned. This highlights the severe health risks posed by informal ULAB recycling, in particular in developing countries, and emphasizes the need to strengthen national and international efforts to address this global public health problem.

Lead is a toxic metal whose widespread use has caused extensive environmental contamination and health problems in many parts of the world. Lead exposure accounts for “almost 1% of the global burden of disease, with the highest burden in developing regions” (Fewtrell et al. 2004). Measures to reduce and control lead use and prevent human exposure to lead, in particular for children, have been put in place at national and international levels [Meyer et al 2008; U.S. Centers for Disease Control and Prevention (CDC) 2005]. However, serious lead intoxications still occur from different sources, in particular in developing countries, posing a major health and environmental challenge (Falk 2003).

Between November 2007 and March 2008, a cluster of 18 deaths caused by a severe and rapidly progressive central nervous system disease of unexplained origin was identified in young children living in the NGagne Diaw neighborhood of Thiaroye sur Mer, in the suburbs of Dakar, Senegal. Local health authorities conducted initial investigations to identify the cause of the outbreak. Differential diagnoses included cholera, meningitis, and cerebral malaria, as these conditions are prevalent at that time of the year; however, investigations disproved these diagnoses. Lead intoxication was then considered because the mothers of some of the children were engaged in the recycling of used lead-acid batteries (ULAB) and the recovery of lead particles from contaminated sand taken from the battery breaking areas. Initial investigations conducted by the Dakar Poisons Centre detected very high blood lead levels in 71 siblings and mothers of the deceased children. Concerned about these findings, the Senegalese Ministry of Health requested the assistance of the World Health Organization (WHO) in investigating and responding to this incident. In this article we describe this investigation.

## Materials and Methods

### Strategic approach

Because autopsies and postmortem testing on the 18 deceased children were not possible for sociocultural reasons, the investigation centered first on the examination of siblings of the deceased children, and their mothers. In a second step, another group of children and adults, living in the same community but unrelated to the deceased children, were also investigated to evaluate the extent of lead intoxication in the area. Environmental health investigations were conducted in parallel to assess environmental contamination and exposure pathways.

### Selection of study participants

All siblings of the 18 deceased children and all of the siblings’ mothers (exact number unknown) were invited by the local health authorities to participate in the study. A total of 32 siblings and 23 siblings’ mothers agreed to participate. A second group of 18 children and 8 adults was selected with the assistance of local community leaders using the following selection criteria: They were not related to the deceased children, they were living in the NGagne Diaw neighborhood, and their age and sex had to be equally distributed. It was not possible to match study groups. All study participants or their representatives gave written consent to participate in the study. The study was approved by the Senegalese national ethics committee.

We considered all 18 children from the NGagne Diaw neighborhood who died from a rapidly progressive central nervous system disease of unexplained origin between November 2007 and March 2008 to be possible victims of lead intoxication.

For the purpose of this study, we defined “children” as ≤ 19 years of age, which corresponds to the WHO definition of “child” and “adolescent.”

### Data collection

The following data were recorded for all 81 individuals investigated: name, age, sex, clinical and neurologic evaluation, medical and occupational histories, global positioning system (GPS) coordinates of their place of residence, and results of laboratory analysis.

We collected epidemiologic and medical information on the 18 children who died between November 2007 and March 2008 from local medical staff or mothers. Data included name, age, sex, name of mother, date and place of death, signs and symptoms, course of illness, and GPS (global positioning system) coordinates of the place of residence.

### Neurologic and clinical investigation

All 81 individuals selected were given a clinical neurologic examination, and their medical and occupational histories were taken. Clinical examinations included a physical examination; measure ment of height, weight, systolic blood pressure, and pulse rate; and abdominal examination. Neurologic investigations included assessment of mental status, motor and sensory systems, and tendon reflexes.

### Collection of blood samples and laboratory analysis

We collected two venous blood samples for each individual. Particular care was taken to prevent external contamination of specimens, including through the meticulous cleaning of venipuncture site. One sample was collected in an EDTA tube for lead analysis. Another sample was collected in a dry tube and analyzed for full blood count, serum iron, creatinine, and transaminases. Lead was analyzed by graphite furnace atomic absorption spectrometry using a PerkinElmer AA600 spectrometer (PerkinElmer, Waltham, MA, USA) by the centralized certified Pasteur Cerba laboratory (Cergy-Pontoise, France). Depending on the lead concentration, blood samples were diluted 5, 10, or 20 times with a diluent and matrix modifier. Other biochemical analyses were performed by the BIO-24 laboratory (Dakar, Senegal). The full blood count was conducted with a Sysmex XT 2000i/1800i analyzer (Sysmex Corporation, Kobe, Hyogo, Japan). Serum iron, creatinine, and transaminases were measured with a Cobas Integra 6000 analyzer (Roche Diagnostics, Basel, Switzerland) using colorimetry, enzymatic colorimetry, and kinetic ultraviolet methods, respectively.

### Environmental investigations

We interviewed local residents, authorities, and medical personnel to gain a better understanding of the context in which the cluster of deaths occurred and the way ULAB recycling practices were conducted before the deaths. Questionnaire-supported interviews of the parents or relatives of 4 deceased children and 10 children with confirmed high blood lead levels were conducted to assess exposure pathways to lead, both during the period when deaths occurred and at the time of our investigation.

We measured environmental lead concentrations *in situ* without sample preparation with a portable X-ray fluorescence analyzer InnovX Alpha (Innov-X Systems, Inc., Woburn, MA, USA) using soil mode. This allows the determination of lead concentration over a surface area of approximately 1 cm^2^ to a depth of approximately 5 mm, which corresponds to the surface soil most accessible to humans. A total of 194 outdoor and 40 indoor concentrations were measured. Lead concentrations of outdoor soil, consisting principally of sand, were measured systematically every 30–50 m along the streets and communal areas throughout the area where the 18 deaths occurred, and every 50–100 m outside of this area. Additional measurements were conducted in areas of particular interest, such as sites where intensive recycling had taken place and areas where children were seen playing with the sandy soil. Indoor concentrations were measured in various locations inside four houses, including on the floor, on furniture, and on bed sheets and mattresses. All GPS coordinates were recorded.

## Results

Epidemiologic, clinical, and laboratory data are presented in Table 1.

### Epidemiology

The 81 individuals (27 males, 54 females) examined as part of the outbreak investigation, comprising 32 siblings of the deceased children, 23 mothers of the siblings (polygamy accounts for the fact that the number of mothers of siblings is greater than that of deceased children), and another 18 children and 8 adults, were all living in the area where the 18 children died. In total there were 50 children 3 months to 19 years of age, and 31 adults 20–64 years of age.

**Figure 1:**
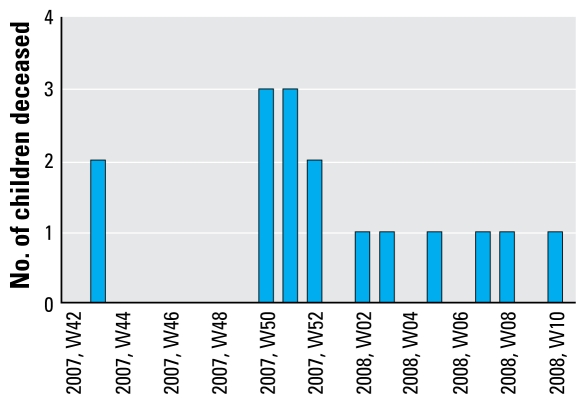
Number of children deceased per calendar week in the NGagne Diaw neighborhood of Thiaroye sur Mer from November 2007 to March 2008.

**Figure 2:**
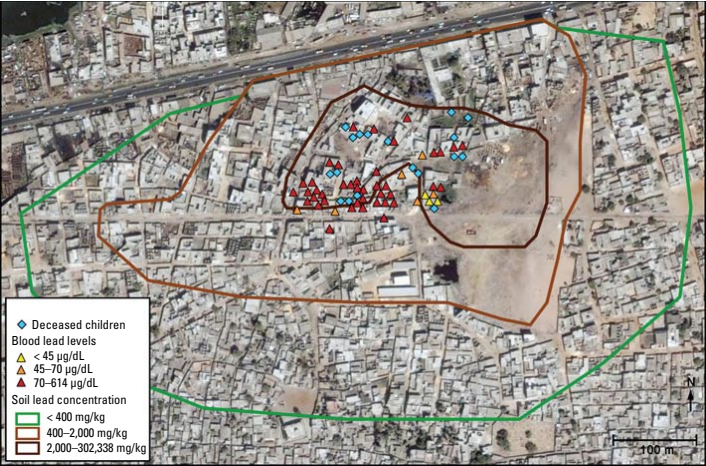
Map of the NGagne Diaw neighborhood of Thiaroye sur Mer, Senegal, showing the geographic distribution of the 18 deceased children, the blood lead levels of the 50 children investigated, and the environmental lead contamination, extrapolated from 194 outdoor measurements. The scale used for the environmental contamination is based on French recommendations for residential and industrial areas set at 400 mg/kg and 2,000 mg/kg, respectively (Laperche et al. 2004).

The 18 children (7 males, 11 females) who died between November 2007 and March 2008 were between 1 and 6 years of age, lived in the NGagne Diaw neighborhood of Thiaroye sur Mer, and suffered from a rapidly progressive central nervous system disease of unexplained origin. Figure 1 shows the number of these children who died per calendar week. The geographic distribution of these 18 deaths, overlaid with other clinical and environmental data, is shown in Figure 2. According to information provided by the Senegalese Agency for Statistics and Demography, an estimated 950 individuals, among them approximately 150 children between 1 and 6 years of age, were living in this area during the epidemic (Agence Nationale de la Statistique et de la Démographie, unpublished data). The 18 deaths therefore equated to a mortality rate of about 12% among resident children of that age. Three of the children died at home and 15 in the hospital.

### Symptoms

Among the 50 children we investigated, we found a high incidence of gastrointestinal (42%) and neuropsychiatric (34%) disorders. The most frequent neuro-psychiatric disorders were irritability, anxiety, sleep disturbance, and frequent crying, reported in 14% of children. Behavioral and sociability disorders were reported in 12% of the children, predominantly in those under 5 years of age. These disorders included aggression, anxiety in playing with other children, and clinginess to the mother. Older children complained of headache. Almost one-third (32%) of children suffered from anorexia or, in the case of small children, difficulty eating. Three children (7, 15, and 20 months of age) refused all food and would only breast-feed. Other gastrointestinal symptoms included colic (18%) and vomiting (10%). We found a history of hospitalization for one or more episodes of convulsions of unexplained cause between September 2007 and June 2008 in 24% of the children overall and in 34% of those who were siblings of the deceased children. The majority of episodes of convulsions (73%; data not shown) occurred between December 2007 and March 2008, during the same period when the deaths were observed.

Of the 31 adults examined, the most frequently reported symptoms were gastrointestinal upset (19%), including epigastric pain and colic, and neuro psychiatric disorders (10%) such as irritability, anxiety, and headache.

Limited clinical information was available on the 18 children who died before an epidemic was suspected. Information provided by local doctors, parents, or relatives indicated that the children had developed acute gastrointestinal illness, including vomiting, bloody diarrhea, and colic, which progressed within hours to days to irritability, convulsions, reduced level of consciousness, coma, and death.

### Signs

On physical examination, 6 (12%) of the 50 investigated children showed apathy, somnolence, or lethargy. Nine (18%) had a positive Babinski sign, reduced rotular reflexes, and altered muscle tone (hyper-tonia or hypotonia). Among the 28 children < 5 years of age, 6 (21%) showed either delayed psychomotor development or regression. Four children showed a significant degree of regression, with loss of sphincter control, loss of ability to walk, and loss of acquired speech. Many children showed low weight and height for their age, but no physical signs of malnutrition were observed. All children had normal blood pressure. No gum or dental abnormalities attributable to lead exposure were observed.

The only abnormalities found on physical examination of the adults were a positive Babinski sign and absent rotular reflexes in 3 of the 23 siblings’ mothers.

### Laboratory results

#### Hematology

Most (80%) of the 50 children investigated were anemic, and half of those had microcytic anemia characterized by alterations of the hemoglobin, hematocrit, and globular volume. Iron deficiency was observed in only 7 children (14%). Of the adults, 13% had anemia, with 10% having microcytic anemia. Anemia was associated with iron deficiency in all adults.

#### Biochemistry

Most (58%) of the children showed a slight to moderate elevation of hepatic enzymes [AST (aspartate amino-transferase) and ALT (alanine amino-transferase)]. Three children showed a slight increase in creatinine concentration, indicating that kidney function might have been slightly affected. Five adults had a slight to moderate elevation of hepatic enzymes (AST and ALT), only one woman (42 years of age) had an increased creatinine concentration.

#### Blood lead levels

In children, the mean (± SD) blood lead concentration was 138.0 ± 60.4 μg/dL (range, 59.1–345.4 μg/dL) for the 32 siblings of deceased children and 114.3 ± 132.5 μg/dL (range, 39.8–613.9 μg/dL) for the 18 children who were unrelated to the deceased children. In adults, the mean (± SD) blood lead concentration was 55.3 ± 19.8 μg/dL (range, 32.5–98.8 μg/dL) for the mothers of the 23 siblings, and 55.9 ± 17.8 μg/dL (range, 37.7–81.0 μg/dL) for the 8 adults who were not related to the deceased children. The blood lead levels measured in all investigated children were mapped, together with additional environmental data and geographic distribution of the deceased children, and are shown in Figure 2.

### Complex lead recycling activities

Informal lead recycling from ULAB has taken place in NGagne Diaw since 1995, concentrated in an open, sandy area of about 40,000 m^2^ (visible in Figure 2). Used batteries from cars, trucks, and other appliances were collected around the city of Dakar and brought to NGagne Diaw to be broken apart. Most of the metallic lead was recovered, whereas other components of the batteries were discarded. Different lead compounds, including elemental lead, lead oxide,and other residues, accumulated in the sandy soil over time. Around October 2007, local people realized that this soil contained commercially profitable lead and started to collect and transport the contaminated soil into the community, sometimes into houses, and to sieve it and extract the larger lead particles. Children were often in proximity to these sieving operations, in some cases even on their mothers’ backs [see Supplemental Material, Figure 1 (doi:10.1289/ehp.0900696.S1 available via http://dx.doi.org/)]. Lead particulate soil was then packed into bags (see Supplemental Material, Figure 2 (doi:10.1289/ehp.0900696. S1)] and sold to a local scrap dealer. A large number of people gradually became involved in this lucrative activity. Toward the end of 2007, the usual buyer stopped coming to NGagne Diaw neighborhood. However, the battery breaking and lead extraction activities did not stop, and the population started to store contaminated soil inside their homes, sometimes even under their beds. Lead recuperation from ULAB and soil in Thiaroye sur Mer is reported to have stopped in March 2008 after a public awareness campaign about the hazards of lead. By that time, however, the contaminated sandy soil had been extensively dispersed throughout the neighborhood, including inside a large number of homes. In March 2008, the Senegalese authorities undertook partial decontamination activities by removing 300 tons of lead-contaminated soil and sacks of lead ingots from the ULAB breaking area and from people’s homes and by covering part of the area with clean sand.

### Environmental contamination

Measurements performed *in situ* in June 2008 in 56 indoor locations revealed lead concentrations up to 14,000 mg/kg inside houses, in particular on floors and mattresses. Measurements performed in another 194 outdoor locations throughout the NGagne Diaw neighborhood revealed soil lead concentrations up to 209,000 mg/kg in the large open, sandy area where ULAB recycling activities had taken place since 1995. Concentrations up to 182,000 mg/kg were measured in residential areas, even in some areas that had been covered with clean sand in March 2008. Bags of soil containing up to 302,000 mg/kg were found throughout the community [see Supplemental Material, Figure 3 (doi:10.1289/ehp.0900696.S1)]. The soil lead concentrations were significantly lower outside the areas where soil sieving and lead extraction activities had taken place, indicating that the lead contamination was geographically limited.

Figure 2 depicts a schematic geographic mapping of the soil contamination extrapolated from the 194 outdoors measurements, together with clinical and analytical data described earlier. The scale is based on French recommendations for residential and industrial areas set at 400 mg/kg and 2,000 mg/kg, respectively (Laperche et al. 2004).

### Exposure pathways

The main lead exposure pathway was most likely through inhalation and/or ingestion of the heavily contaminated sandy soil and dust in suspension. This occurred during the period that recycling was being carried out and continued after recycling was stopped because of the high level of residual contamination. Young children were particularly exposed by ingestion through hand-to-mouth behavior and some pica while playing outdoors on contaminated soil or with the lead-rich soil stored inside their homes. This was corroborated during the field investigations when numerous children were observed playing with and ingesting the contaminated sandy soil, indicating that exposure was still ongoing at the time of our investigation [see Supplemental Material, Figure 4 (doi:10.1289/ehp.0900696.S1)].

## Discussion

Lead is a cumulative toxicant that affects multiple body systems, including the neurologic, hematologic, gastrointestinal, cardiovascular, and renal systems. The toxic effects of lead range from abdominal pain, loss of appetite, vomiting, and anemia to irritability, ataxia, stupor, coma, seizures, and death. Children are particularly vulnerable to the neurotoxic effects of lead because a greater proportion of systemically circulating lead enters their brains, and the developing nervous system is more susceptible to the toxic effects of lead than the mature brain. In addition, children are more likely to be exposed to environmental sources because of their hand-to-mouth behavior and the higher absorption of ingested lead in the gastrointestinal tract (up to 50%) compared with adults (20–30%) (Lidsky and Schneider 2003). Children can also be exposed prenatally because lead crosses the placenta. Because of the special susceptibility of children, even relatively low levels of exposure can cause serious and in some cases irreversible neurologic damage, leading to permanent intellectual impairment (Dart et al. 2004). Blood lead concentrations < 10 μg/dL have been associated with cognitive impairment, and recent evidence suggests that there may be no safe level (Bellinger et al. 2003; Canfield et al. 2003; Chiodo et al. 2004; Kordas et al. 2006; Lanphear et al. 2000, 2005; Tellez-Rojo et al. 2006). The severity of signs and symptoms increases with exposure, and in children blood lead levels > 70 μg/dL are generally associated with life-threatening toxicity, including coma and convulsions. Treatment of childhood lead poisoning is determined by an assessment of the clinical condition of the patient together with the blood lead level. As a guide, the CDC (2002) recommends that, in children, blood lead levels > 10 μg/dL require further monitoring; levels > 20 μg/dL require further monitoring, environmental investigation and control; levels > 45 μg/dL require clinical evaluation and chelation therapy; and those > 70 μg/dL require immediate hospitalization and chelation therapy. Importantly, chelation therapy must always be conducted in a lead-free environment. In all cases, removal of the source of lead exposure is the mainstay of management.

Our clinical and laboratory investigations revealed that all 81 individuals studied were poisoned, often severely, with lead. This group included persons who had no direct link with the deceased children and individuals who were never involved in lead recycling or sieving activities. Of the 50 children investigated, 17 showed signs and symptoms of severe chronic lead poisoning, in particular neurologic, developmental, and behavioral disorders. Four children < 5 years of age showed severe regression of psychomotor development. Almost all of the children (94%) had blood lead levels > 45 μg/dL, requiring chelation therapy to limit short- and long-term neurodevelopmental and health consequences. Moreover, 41 children (82%) had life-threatening blood lead levels > 70 μg/dL, therefore requiring urgent medical treatment. The highest blood level, which was measured by a centralized certified laboratory, was 613.9 μg/dL in a 20-month-old boy. To the best of our knowledge, this is the highest blood lead level ever recorded in a living child of this age. Remarkably, this child did not show overt signs of encephalopathy, although he was underweight, apathetic, anorexic, and anemic, and at the time of our investigation would only take breast milk. Although this child’s mother was not herself involved in lead recycling, sieving of soil was performed outside her house, and lead-enriched soil was stored in her home by other residents. These results suggest that other habitants of the affected area—estimated at 950, including about 460 children < 19 years of age—are also likely to be poisoned with lead. Among these persons, young children and those yet to be born are of particular concern because of their special vulnerability.

Lead concentrations as high as 302,000 mg/kg were measured in outdoor soil, whereas concentrations up to 14,000 mg/kg (on a mattress) were detected indoors. These values exceed guidelines from France and the United States for residential areas set at 400 mg/kg by several orders of magnitude (Laperche et al. 2004; U.S. Department of Housing and Urban Development 1995). These results indicate that despite decontamination activities undertaken in March 2008 by the Senegalese authorities, the neighborhood was still heavily contaminated. Our investigation showed that the most severe environmental contamination (up to 302,000 mg/kg) was limited to the area where ULAB had been recycled over many years and to the residential area where sieving and storage of contaminated soil had taken place between October 2007 and February 2008, corresponding to the area depicted by the dark brown line in Figure 2. The immediate vicinity of this zone, depicted by the light brown line in Figure 2, was also contaminated, but to a lesser extent (400–2,000 mg/kg).

Our environmental health investigations suggested that the individuals studied had been exposed through inhalation and/or ingestion of the heavily contaminated sandy soil or the dust in suspension. Exposure occurred during the period when recycling was being carried out and continued after recycling was stopped because of the high level of residual contamination. This is supported by the geographic overlap between the areas of high environmental lead contamination and the locations of the homes of the deceased children and of those with high blood lead levels (Figure 2). The extremely high blood lead levels measured in some young children are most likely attributable to hand-to-mouth behavior and some pica while playing in such a heavily contaminated environment. Because this behavior is developmentally normal for young children, it highlights the importance of ensuring that children have a lead-free environment in which to live, play, and learn. The findings of our environmental health investigations further support our suspicion that other inhabitants of the contaminated area are also likely to be poisoned with lead.

The severity of this incident, in terms of environmental contamination and consequent intoxication of a community, is unusual and arose from the particular way in which the informal recycling was carried out in NGagne Diaw. This involved not only the breaking up and melting of metallic lead from ULAB but also the transport, sieving, and storage of lead particles, which were released into the sandy soil during battery recycling. This particular type of activity, which amounted to the recycling of third-hand lead waste, has rarely been observed, even in the developing world.

We were not able to include a control group in the investigation because of the high degree of local sensitivity about the incident. In addition, the investigation was carried out in the context of an emergency response and focused on an initial assessment with a view to identifying the immediate management needs and making recommendations for further action. However, background levels of blood lead among Senegalese children were estimated in 2006 by Diouf et al. (2006). They found that the mean lead level (± SD) of 168 children 8–12 years of age who had lived since birth in Dakar was 10.0 ± 3.9 μg/dL, with a range of 3.1–22.0 μg/dL. Of these children, 99 (58.9%) had blood lead levels > 10 μg/dL. It is therefore possible that the children living in NGagne Diaw neighborhood could already have been suffering from subclinical lead poisoning before this incident occurred. However, blood lead concentrations measured during our investigations were significantly higher than those expected for children living in Dakar.

The diagnosis of the 18 children who died between October 2007 and March 2008 could not be confirmed because medical documentation of the cases was limited; blood samples were not collected for lead analysis; and post mortem investigations could not be conducted for sociocultural reasons. It is notable, however, that 11 of the 32 siblings investigated were hospitalized for convulsions during that time period. In June 2008 the mean blood lead level of the 32 siblings was 138.0 μg/dL. Circumstantial evidence, including the high blood lead levels in siblings and heavy environmental contamination, together with the verbal descriptions of the course of illness provided by family members and physicians, suggests that most, if not all, of the 18 cases of fatal neurologic illness reported between October 2007 and March 2008 were due to encephalopathy resulting from severe lead intoxication (Dart et al. 2004; Henretig 2006).

The methodology we used to investigate this disease outbreak allowed the rapid assessment of both the extent of the environmental contamination and its health consequences on the exposed population. This was crucial, as it enabled risk mitigation and management measures to be taken immediately. In particular, the simple *in situ* X-ray fluorescence methodology used allowed the real-time estimation of lead contamination throughout the contaminated area. Although this method is subject to some inaccuracy arising from various factors such as soil hetero genicity, lack of sample preparation, and instrument imprecision, this is acceptable in view of the extremely high levels measured and the purpose of these measurements (U.S. Environmental Protection Agency 2007). Finally, the lead intoxication could have been detected earlier if blood samples of the deceased children had been collected and stored, at the time of their hospitalization, for further diagnostic testing.

## Conclusions

Our study revealed a mass lead intoxication resulting from informal and unsafe ULAB recycling activities. Despite international and national efforts to prevent human exposure to lead, serious mass lead poisonings with severe health consequences still occur. Lead contained in car batteries is of particular concern because it accounts for an estimated 80% (6,975,000 metric tons in 2008) of worldwide lead consumption and generates large quantities of lead waste in virtually every country (International Lead and Zinc Study Group 2009a, 2009b). Because lead is a valuable commodity, informal and unsafe lead recycling has become a widespread activity in many developing countries, exposing large populations to the short- and long-term harmful effects of lead. Although lead intoxications from unsafe ULAB recycling have been described in many parts of the world (Kaul and Mukerjee 1999; Matte et al. 1989; Nriagu 1992; Paoliello and DeCapitani 2005; Saraci and Ziegler-Skylakakis 1999; Suplido and Ong 2000), these activities are usually unregulated, and therefore the global magnitude of the problem is not documented. Moreover, it is likely that many incidents remain unnoticed or unaddressed because of insufficient awareness of the problem or limited capacities in developing countries for their detection and management.

Lead intoxication is a preventable environmental illness, and efforts must be taken at all levels to protect populations. Relevant national interventions include the implementation and enforcement of regulations, legislation, and international guidelines and conventions governing the use and recycling of lead and lead-based products; public education about the health hazards of lead; and promotion of environmentally sound ULAB recycling (Basel Convention 2003; United Nations Environment Programme 2008; WHO 1995). At the international level, conventions and agreements on chemical safety should be revisited and strengthened to address the negative public health impact that could result from informal lead recycling. Parallel to primary prevention efforts, capacities to detect and manage lead exposures also need to be strengthened to allow the effective identification of and response to incidents when they occur and also to identify populations at risk and therefore help to prioritize and target interventions. Global prevention of lead intoxication requires coordination and cooperation, both at national and international levels, between a number of stakeholders, including the health and environment sectors, regulatory and law enforcement bodies, industry, and civil society. In the case of many developing countries, national efforts will also need the technical and financial support of donor organizations and international agencies.

## Figures and Tables

**Table 1:** Epidemiologic, clinical, and analytical data for the 81 individuals investigated.

	Siblings of deceased children	Siblings’ mothers	Other children	Other adults	Total	Total children	Total adults
No. of individuals	32	23	18	8	81	50	31
Age							
Range	7 months–7 years	20–44 years	3 months–19 years	20–64 years	3 months–64 years	3 months–19 years	20–64 years
Mean ± SD	3 ± 2 years	32 ± 7 years	9 ± 6 years	38 ± 15 years	16.0 ± 15.5 years	5.2 ± 4.6 years	33.4 ± 10 years
Sex (male/female)	14/18	0/23	9/9	4/4	27/54	23/27	4/27
Gastrointestinal disorders (no.)	13	5	8	1	27	21	6
Anorexia	12	0	4	0	16	16	0
Colic	3	4	6	1	14	9	5
Vomiting	5	1	0	0	6	5	1
Neuropsychiatric disorders (no.)	13	1	4	2	20	17	3
History of convulsions	11	0	1	0	12	12	0
Irritability, anxiety, sleeping disorders	4	1	3	2	10	7	3
Behavioral and sociability disorders	3	0	3	0	6	6	0
Other symptoms (no.)							
Headache	1	6	2	0	9	3	6
Neurologic signs (no.)	9	3	3	0	15	12	3
Apathy, lethargy	3	0	3	0	6	6	0
Abnormal examination (rot/Babinski, tone)	9	3	NA	NA	12	9	3
Psychomotor retardation	4	0	2	0	6	6	0
Major regression	2	0	2	0	4	4	0
Hematology (no.)							
Anemia	29	3	11	1	44	40	4
Microcytic	18	3	6	0	27	24	3
Iron deficiency	3	3	4	1	11	7	4
Biochemistry							
Creatinine concentration (no.)							
Decreased	14	0	7	1	22	21	1
Elevated	2	1	1	0	4	3	1
Normal	16	22	10	7	55	26	29
Liver enzymes							
AST concentration (no.)							
Elevated	26	4	7	0	37	33	4
Normal	6	19	11	8	44	17	27
ALT concentration (no.)							
Elevated	1	4	3	0	8	4	4
Normal	31	19	15	8	73	46	27
Blood lead levels							
Range (μg/dL)	59.1–345.4	32.5–98.8	39.8–613.9	37.7–81.0	32.5–613.9	39.8–613.9	32.5–98.8
Mean ± SD (μg/dL)	138.0 ± 60.4	55.3 ± 19.8	114.3 ± 132.5	55.9 ± 17.8	101.1 ± 81.7	129.5 ± 92.4	55.5 ± 19.0
> 45 μg/dL (no.)	32	14	15	5	66	47	19
> 70 μg/dL (no.)	31	5	10	2	48	41	7

Abbreviations: NA, not available; rot, rotular reflex. “Children” has been defined as ≤ 19 years of age.
